# ISG15 deficiency and increased viral resistance in humans but not mice

**DOI:** 10.1038/ncomms11496

**Published:** 2016-05-19

**Authors:** Scott D. Speer, Zhi Li, Sofija Buta, Béatrice Payelle-Brogard, Li Qian, Frederic Vigant, Erminia Rubino, Thomas J. Gardner, Tim Wedeking, Mark Hermann, James Duehr, Ozden Sanal, Ilhan Tezcan, Nahal Mansouri, Payam Tabarsi, Davood Mansouri, Véronique Francois-Newton, Coralie F. Daussy, Marisela R. Rodriguez, Deborah J. Lenschow, Alexander N. Freiberg, Domenico Tortorella, Jacob Piehler, Benhur Lee, Adolfo García-Sastre, Sandra Pellegrini, Dusan Bogunovic

**Affiliations:** 1 Department of Microbiology, Icahn School of Medicine at Mount Sinai, New York 10029, USA; 2 Institut Pasteur, Cytokine Signalling Unit, CNRS URA 1961, 75724 Paris, France; 3 Department of Biology, University of Osnabrück, 49076 Osnabrück, Germany; 4 Immunology Division, Hacettepe University, Ihsan Dogramaci Children's Hospital, 06100 Ankara, Turkey; 5 Division of Infectious Diseases and Clinical Immunology, Pediatric Respiratory Diseases Research Center, National Research Institute of Tuberculosis and Lung Diseases, Shahid Beheshti University of Medical Sciences, 4739 Teheran, Iran; 6 Department of Medicine and Department of Pathology and Immunology, Washington University School of Medicine, St. Louis, Missouri 63110, USA; 7 Department of Pathology, University of Texas Medical Branch, Galveston, Texas 77555, USA

## Abstract

ISG15 is an interferon (IFN)-α/β-induced ubiquitin-like protein. It exists as a free molecule, intracellularly and extracellularly, and conjugated to target proteins. Studies in mice have demonstrated a role for Isg15 in antiviral immunity. By contrast, human ISG15 was shown to have critical immune functions, but not in antiviral immunity. Namely, free extracellular ISG15 is crucial in IFN-γ-dependent antimycobacterial immunity, while free intracellular ISG15 is crucial for USP18-mediated downregulation of IFN-α/β signalling. Here we describe *ISG15*-deficient patients who display no enhanced susceptibility to viruses *in vivo*, in stark contrast to *Isg15*-deficient mice. Furthermore, fibroblasts derived from *ISG15*-deficient patients display enhanced antiviral protection, and expression of ISG15 attenuates viral resistance to WT control levels. The species-specific gain-of-function in antiviral immunity observed in ISG15 deficiency is explained by the requirement of ISG15 to sustain USP18 levels in humans, a mechanism not operating in mice.

## Abstract (web summary)

ISG15 is a ubiquitin-like protein which has important immune-related functions in mice and humans. Here the authors demonstrate that, unlike in mice, human ISG15 stabilizes UPS18 and that ISG15-deficient human cells are more resistant to viral infection.

## Graphical abstract

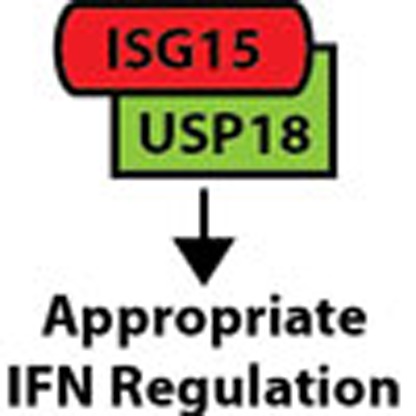

Interferon-stimulated gene 15 (ISG15) is an interferon (IFN)-α/β-inducible ubiquitin-like molecule. It has two ubiquitin-like domains and is synthesized from an immature precursor by cleavage of a C-terminal extension peptide^1^. Mature ISG15 has the LRLRGG sequence, which is also present at the C terminus of mature ubiquitin. ISG15 exists in two distinct states: as a free molecule (intracellular and extracellular) or conjugated to target protein lysine residues (ISGylation)^2^,^3^,^4^,^5^,^6^,^7^,^8^,^9^,^10^. In a process similar to ubiquitin ligation, ISGylation involves a conjugation pathway including E1 (UBE1L), E2 (UBCH8) and E3 (HERC5, EFP, HHARI) enzymes. ISGylation is reversed by the action of the isopeptidase ubiquitin-specific protease 18 (USP18; UBP43)^11^,^12^. Accordingly, cells deficient in the conjugation machinery exhibit absent or reduced ISGylation, while *Usp18*-deficient cells exhibit high levels of Isg15 conjugates^13^. All of the enzymes involved in ISGylation/de-ISGylation are transcriptionally induced by IFN-α/β.

Numerous *in vivo* and *in vitro* studies have ascribed an antiviral role to ISG15. Mice lacking Isg15 exhibit enhanced susceptibility to challenge with multiple viruses, including murine gammaherpesvirus 68 (ref. 14), influenza A virus (IAV)^14^, influenza B virus^14^,^15^, Sindbis virus^14^, vaccinia virus^16^, herpes simplex virus 1 (HSV-1)^14^, Chikungunya virus^17^ and murine norovirus^18^, yet no enhanced susceptibility to vesicular stomatitis virus (VSV) and lymphocytic choriomeningitis virus^19^. *In vitro* studies in mouse embryonic fibroblasts (MEFs), mouse lung fibroblasts and RAW 264.7 cells have demonstrated an antiviral role for Isg15 during Sindbis virus^20^, vaccinia virus^16^, Ebola virus^21^, Dengue virus and West Nile virus^22^ infection, although there are several reports of viruses exhibiting no enhanced replication in the absence of Isg15 (murine gammaherpesvirus 68, HSV-1, Sindbis virus, IAV^14^). ISG15 small interfering RNA (siRNA) knockdown in human cells has also suggested an antiviral role for free and/or conjugated ISG15 during infection with numerous viruses, including IAV^23^, human immunodeficiency virus^24^,^25^,^26^, Ebola virus^21^,^27^, human papilloma virus^28^, VSV^27^, Japanese encephalitis virus^15^ and Sendai virus (SeV)^29^. While these many studies have suggested an antiviral role for ISG15, others have suggested no role at all; the one clear exception is hepatitis C virus (HCV). *In vitro* studies of HCV infection suggest that ISG15 exhibits proviral activity^30^,^31^. The silencing of ISG15 in human hepatoma cells was found to lead to an inhibition of HCV replication, increases in cell sensitivity to IFN-α/β and accumulation of IFN-α/β-stimulated gene products (ISGs).

Our studies on *ISG15*-deficient individuals have demonstrated a critical role for free extracellular ISG15 in the induction of IFN-γ and protection against mycobacterial disease. These findings were confirmed in *Isg15*-deficient mice^9^. Intriguingly, there is no evident overlap in viral susceptibility phenotype between mice and humans lacking ISG15. Despite being serologically positive for numerous childhood viruses, such as human cytomegalovirus (HCMV), Epstein Barr virus, measles virus, varicella-zoster virus, HSV-1 and -2, mumps virus, IAV and hepatitis A virus, *ISG15*-deficient individuals exhibit no overt susceptibility to viral disease^9^,^10^. We recently demonstrated that human free intracellular ISG15 binds USP18, a negative regulator of IFN-α/β signalling. This binding prevents the SKP2-mediated degradation of USP18, and thus is critical for the accumulation of USP18 and the appropriate regulation of IFN-α/β signalling. Indeed, patients lacking ISG15, and consequently having low USP18 levels, display higher steady-state levels of ISG transcripts in whole blood than controls^10^,^32^.

We demonstrate that human cells deficient in ISG15 exhibit a persistent elevation in ISG expression. We find that, not only do humans deficient in ISG15 lack any overt susceptibility to viral infection, but cells derived from these individuals exhibit an enhancement of broad protection against viruses. Complementation of ISG15-deficient cells with the wild-type (WT) or conjugation-deficient ΔGG *ISG15* alleles rescues both WT levels of ISG expression and viral susceptibility. Furthermore, we demonstrate that there is a species-specific interaction between ISG15 and USP18. While humans require ISG15 for USP18 stability and appropriate negative regulation of the IFN response, mice lack this stable interaction and can regulate the IFN response in the absence of Isg15.

## Results

### Persistent increase of ISG expression in cells lacking ISG15

**Figure 1:**
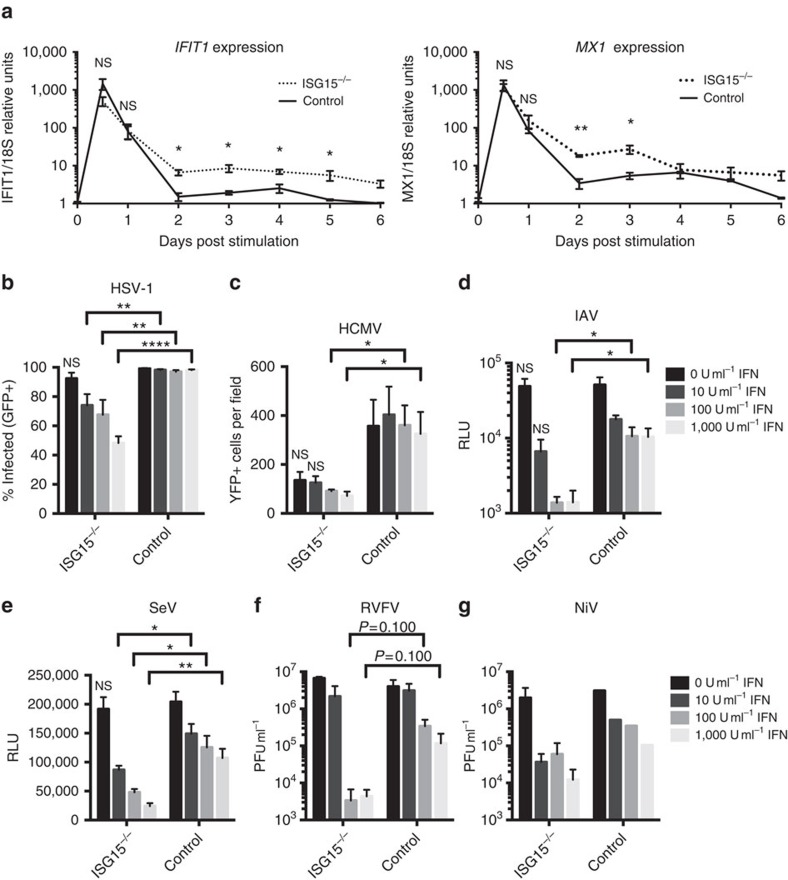
*ISG15*-deficient cells exhibit prolonged ISG expression and resistance to infection with viruses from multiple families. hTert-immortalized fibroblasts from *ISG15*-deficient patients (*n*=3) or controls (*n*=5) were treated with the indicated concentration of IFN-α2b for 12 h, washed, and allowed to rest for 36 h before infection. (**a**) Relative *IFIT1* (left panel) or *MX1* (right panel) mRNA levels at 12 (time of IFN-α2b removal), 24, 48, 72, 96, 120 and 144 h post-priming with 1,000 IU ml^−1^. (**b**) Cells were infected with HSV-1-US11-GFP at a multiplicity of infection (MOI) of 1.0 for 24 h, fixed, subjected to Hoechst 33342 nuclear staining, and imaged. Shown are the percentages of cells positive for both GFP and nuclear staining. (**c**) Cells were infected with HCMV-IE2-YFP at an MOI of 2.0 for 24 h, fixed, and imaged. Shown are the numbers of GFP-positive cells field^−1^. (**d**) Cells were infected with IAV PR8-gLuc-PTV1 at an MOI of 10.0 for 24 h. Lysates were collected and assayed for luciferase activity. Shown are the results in relative luminescence units. (**e**) Cells were infected with SeV-GFP-gLuc at an MOI of 0.1 for 24 h. Supernatants were collected and assayed for luciferase activity. Shown are the results in relative luminescence units. (**f**) Cells were infected with RVFV at an MOI of 0.01 for 48 h. Supernatants were collected and titered by plaque assay. (**g**) Cells were infected with NiV-gLuc-P2A-eGFP at an MOI of 0.01 for 24 h. Supernatants were titered by plaque assay. **a** shows the combined results of two experiments. **b**,**c**, and **e** show a single representative experiments of three performed. **d** shows the combined results of three experiments. **f** and **g** are single experiments performed in the BSL-4. Error bars, s.d. Comparisons made with unpaired *t*-test. *P<0.05, **P<0.01, ****P<0.0001. NS, not significant.

The *ISG15*-deficient individuals identified to date have been infected with numerous childhood viruses, against which they mounted a humoral immune response^9^,^10^. No overt viral illnesses were documented in these patients, suggesting that ISG15 may not be essential for immunity to these viruses. *ISG15*-deficient patients may actually have a milder disease course than wild-type individuals. However, this is difficult to demonstrate, as information about subclinical illness is often not transmitted by patients to their doctors or noted by the doctors themselves. We tested this hypothesis *in vitro* using hTert-immortalized dermal fibroblasts derived from *ISG15*-deficient patients and relevant WT controls. Human cells lacking ISG15 also have low USP18 levels (due to the lack of USP18 stabilization by ISG15) and high levels of ISG transcripts, as documented *ex vivo* with leukocytes from *ISG15*-deficient individuals^10^. We carried out *in vitro* studies, monitoring the kinetics and persistence of ISG induction by quantitative real-time PCR in WT and *ISG15*-deficient fibroblasts during and after IFN priming, in which the cells were pulsed with IFN-α2b for 12 h and then allowed to rest for 36 h. We determined mRNA levels for the IFN-induced protein with tetratricopeptide repeats 1 (IFIT1) and myxovirus resistance 1 (MX1), from 12 h to 6 days post-IFN-α/β priming. The expression levels of *IFIT1* and *MX1* transcripts during peak induction and early dampening were similar between *ISG15*-deficient and control cells (Fig. 1a). However, the time taken for *IFIT1* transcript levels to return to baseline after IFN priming differed between control and *ISG15*-deficient cells. Specifically, *IFIT1* transcript levels remained significantly elevated for more than 5 days in *ISG15*-deficient cells, whereas they returned to basal levels within 2 days in control cells (Fig. 1a).

### *ISG15*-deficient IFN-primed cells are resistant to viral infection

*ISG15*-deficient patients had previously been infected with HSV-1, HCMV and IAV, as indicated by the results of serological tests^10^, but did not develop severe disease. We, therefore, investigated whether the prolonged increase in ISG levels affected the susceptibility of the patients' cells to viral challenge. When IFN-α/β-primed *ISG15*-deficient fibroblasts were infected with HSV-1 (Fig. 1b), HCMV (Fig. 1c) or IAV (Fig. 1d) levels of viral replication were significantly lower in *ISG15*-deficient cells than in control cells. SeV, a murine paramyxovirus known to be highly sensitive to human ISGs, also replicated significantly less efficiently in IFN-α/β-primed *ISG15*-deficient cells (Fig. 1e). Finally, we investigated whether *ISG15*-deficient cells were also resistant to highly pathogenic viruses affecting humans that our patients would have been unlikely to encounter, by infecting control and *ISG15*-deficient fibroblasts with Rift Valley fever virus (RVFV) and Nipah virus (NiV). Both of these viruses also appeared to replicate less efficiently in IFN-α/β-primed *ISG15*-deficient cells than in control cells (Fig. 1f,g), although neither RVFV nor NiV reached statistical significance. Thus, the prolonged induction of ISGs observed in *ISG15*-deficient human cells protects against viruses from multiple families, having both RNA and DNA genomes, and causing both low and highly pathogenic disease in normal healthy humans. These results further suggest that viral infections in *ISG15*-deficient individuals may follow an attenuated course with milder symptoms. Thus, the *in vivo* clinical follow-up of *ISG15*-deficient individuals, serological testing and the results of *in vitro* studies of patient-derived cells all indicate a greater resistance to viral challenge, consistent with a proviral role of ISG15 in humans.

### WT or ΔGG *ISG15* allele restores WT viral susceptibility

**Figure 2:**
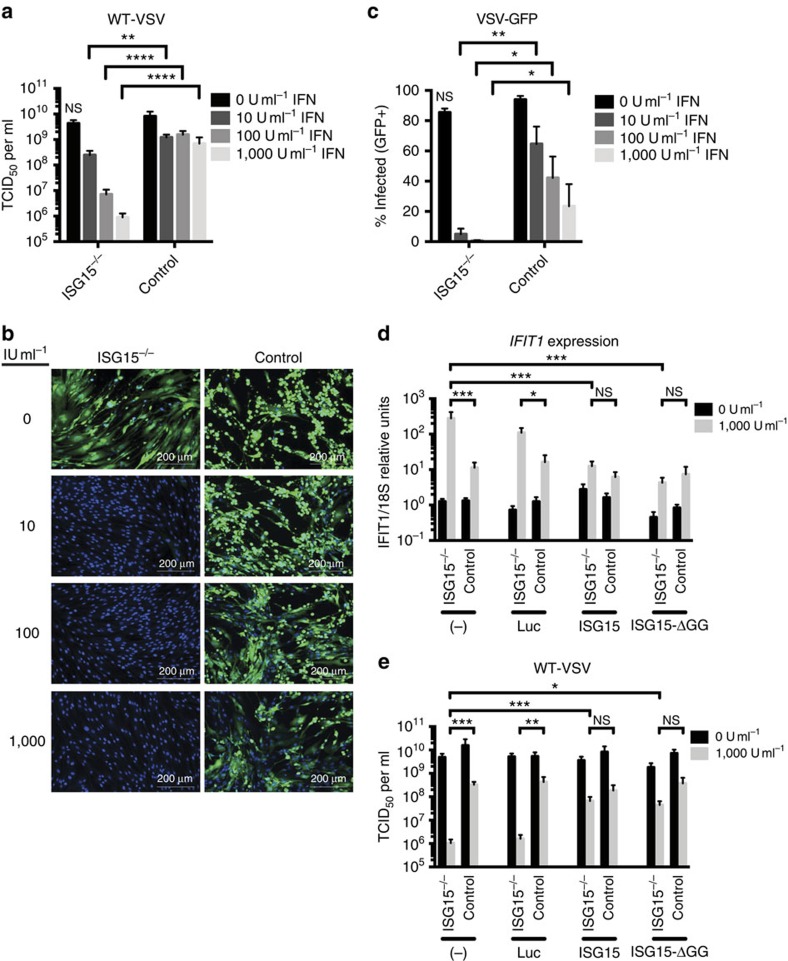
WT ISG15 and ISG15ΔGG restore VSV infection phenotype in *ISG15*-deficient cells. (**a**–**c**) hTert-immortalized fibroblasts from *ISG15*-deficient patients (*n*=3) or controls (*n*=2 or 3) were treated with the indicated concentration of IFN-α2b for 12 h, washed, and allowed to rest for 36 h before infection. (**a**) Cells were infected with VSV at an MOI of 1.0 for 24 h. Supernatants were titered by TCID_50_. (**b**) Cells were infected with VSV–GFP at an MOI of 1.0 for 24 h, fixed, subjected to nuclear staining, and imaged. Representative images are shown for each treatment group. (**c**) Quantification of panel b. Shown is the percentage of cells positive for both GFP and nuclear staining. (**d**) and (**e**) hTert-immortalized fibroblasts from *ISG15*-deficient patients (*n*=3) or controls (*n*=2), untransduced or stably transduced with luciferase, ISG15 or ISG15ΔGG, were mock-treated or primed with 1,000 IU ml^−1^ IFN-α2b for 12 h, washed, and allowed to rest for 36 h. (**d**) Relative *IFIT1* mRNA levels 48 h post-priming. (**e**) Fibroblasts were infected with VSV at an MOI of 1.0, 48 h post-priming. Supernatants were collected at 24 h post-infection and titered by TCID_50_ in duplicate. **a**,**d** and **e** show the combined results of three experiments. **b** and **c** show single representative experiments of three performed. Error bars, s.d. Comparisons made with unpaired *t*-test. *P<0.05, **P<0.01, ***P<0.001, ****P<0.0001. NS, not significant.

We verified that the observed phenotype was due solely to the lack of ISG15 by stably transducing *ISG15*-deficient patient and control fibroblasts with lentiviral particles encoding *ISG15* or control luciferase^10^. The cells were subsequently infected with VSV, another virus known to be sensitive to low levels of IFN-α/β. Control fibroblasts displayed similar levels of VSV replication with and without IFN-α/β priming, suggesting that most of the ISG antiviral activity had declined during the post-priming rest (Fig. 2a). By contrast, IFN-α/β-primed cells deficient in ISG15 displayed significantly lower levels of viral replication (Fig. 2a), indicating a protracted antiviral state, as confirmed with other viral challenges (Fig. 1b-g). The resistance phenotype was confirmed using fibroblasts from the three *ISG15*-deficient patients and five controls (Supplementary Fig. 1). Similar phenotypic differences in viral replication were observed between control and *ISG15*-deficient fibroblasts infected with VSV-expressing GFP (VSV–GFP), as assessed by fluorescence microscopy (Fig. 2b,c). Complementation of *ISG15*-deficient patient fibroblasts with WT ISG15, but not with control luciferase, restored both ISG expression (Fig. 2d) and viral replication (Fig. 2e) to the levels observed in the control cells. We have previously shown that the conjugation-deficient mutant of ISG15 (ISG15ΔGG) retains the ability to bind and stabilize USP18, and is thus a functional negative regulator of IFN signalling^10^. Accordingly, we used *ISG15*-deficient fibroblasts transduced with ISG15ΔGG, which exhibit fully functional downregulation of IFN signalling but no ISGylation, to determine the relative role of each function during viral infection. As observed with WT ISG15, complementation with ISG15ΔGG rescued both the ISG expression and VSV replication phenotypes in *ISG15*-deficient fibroblasts (Fig. 2d,e). Similar results were obtained in A549 and Hela cells that were silenced with siRNA targeting ISG15 or a non-silencing control. In both cell lines, knockdown of ISG15 enhanced ISG expression and resistance to viral replication (Supplementary Fig. 2a-f). These data demonstrate the dependence of the *in vitro* phenotype on the *ISG15* allele and suggest that, in human epithelial cells, the dominant role of ISG15 during viral replication is the ISGylation-independent downregulation of IFN-α/β signalling.

### *Isg15*-deficient mice do not exhibit elevated ISG expression

**Figure 3:**
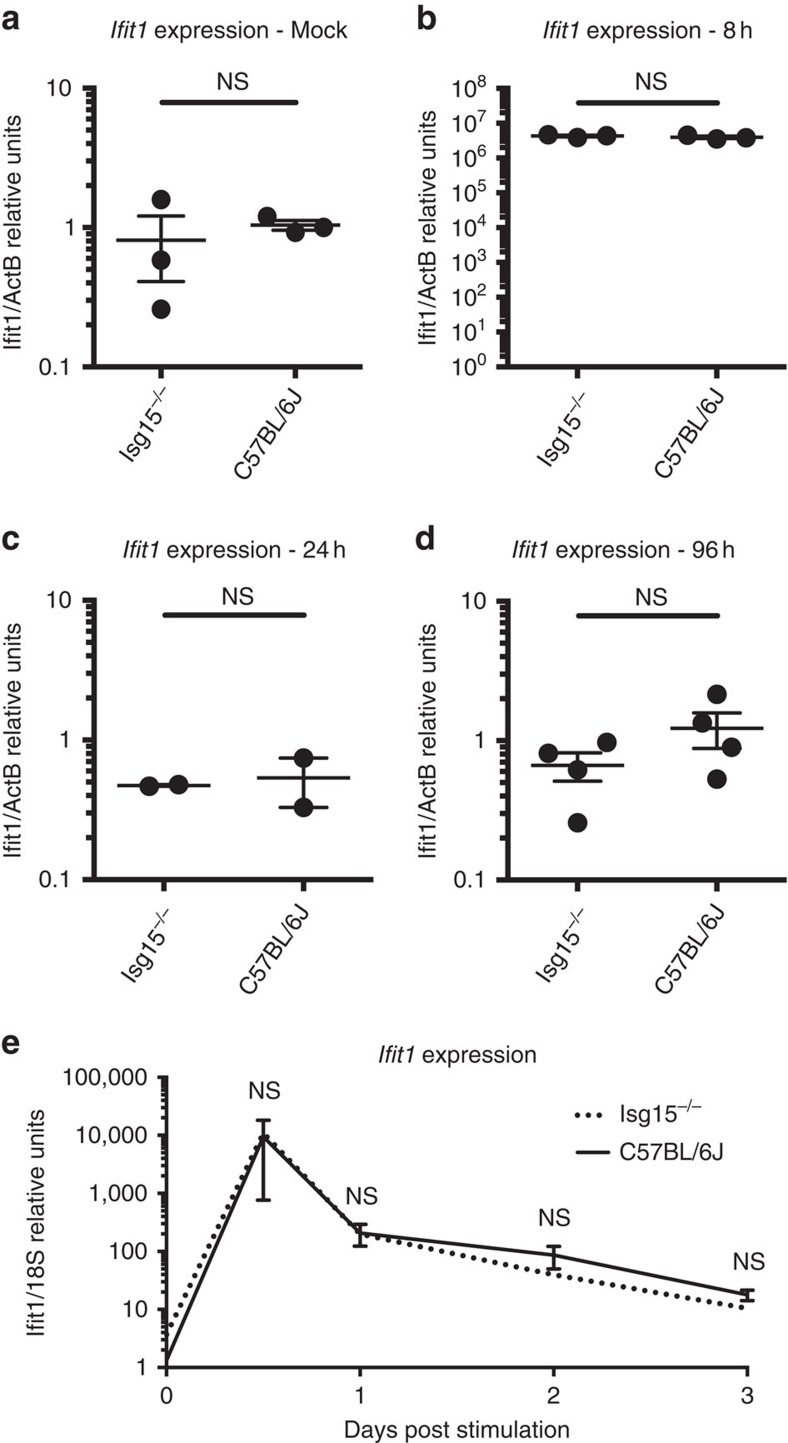
Murine Isg15 deficiency does not affect the regulation of *Ifit1* transcripts. (**a**–**d**) Age-matched *Isg15*-deficient (*n*=2–4 per group) or WT C57BL6/J (*n*=2-4 group^-1^) mice received intraperitoneal injections of (**a**) PBS (mock) or (**b**–**d**) 10,000 IU type-I IFN. Animals were sacrificed at the (**b**) 8 h, (**c**) 24 h or (**d**) 96 h post treatment and relative *Ifit1* mRNA levels were determined by qPCR. (**e**) MEFs derived from *Isg15*-deficient or C57BL/6 J mice were mock-treated or primed with 1000 IU ml^−1^ type-I IFN for 12 h, washed and allowed to rest for 36 h. Relative mRNA levels for *Ifit1* were determined by qPCR at the indicated times post-priming. **e** shows the combined results of three experiments. Error bars, s.d. Comparisons made with unpaired *t*-test. NS, not significant.

All of our observations, whether from the clinical history of *ISG15*-deficient patients or from *in vitro* studies of *ISG15*-deficient patient-derived cells, point to a lack of overt susceptibility to viruses, and suggest that ISG15 may exhibit a proviral role. We were unable to reconcile these data with the numerous reports ascribing a critical antiviral role to Isg15 during the infection of mice *in vivo* with multiple viruses (for example, chikungunya virus^17^, murine gammaherpesvirus 68 (ref. 14), IAV^14^, influenza B virus^14^,^33^, Sindbis virus^14^, vaccinia^16^, HSV-1 (ref. 14) and murine norovirus^18^). We, therefore, analysed whole blood from *Isg15*-deficient mice, to determine whether these mice, like *ISG15*-deficient humans, also displayed elevated levels of steady-state ISG expression in the absence of diagnosable infection^10^. In unstimulated conditions, *Ifit1* mRNA levels of *Isg15*-deficient animals were similar to those of their age-matched WT cagemates (Fig. 3a). This indicates a species-specific difference in ISG regulation in the absence of ISG15. We then hypothesized that, *in natura*, humans are constantly exposed to environmental challenges that induce IFN-α/β, resulting in persistently high levels of ISGs in *ISG15*-deficient individuals, due to a lack of negative regulation. Our mice were not housed in completely sterile conditions, but were nevertheless somewhat shielded from true environmental exposure to microbes. IFN-α/β induction was mimicked by injecting 10,000 U of type-I IFN intraperitoneally into WT control and *Isg15*-deficient mice, followed by analysis of the levels of ISG transcripts in whole blood at multiple timepoints. As expected, the animals receiving IFN injections displayed a robust increase in ISG expression within 8 h that was fully resolved within 24 h. However, no difference in ISG expression levels between *Isg15*-deficient and WT mice was observed at any time point (Fig. 3b–d). For further comparison with our results in human cells, primary MEFs derived from both WT and *Isg15*-deficient mice were primed with type-I IFN for 12 h, allowed to rest for 36 h and the levels of *Ifit1* mRNA were analysed. No differences in *Ifit1* expression levels were observed between WT and *Isg15*-deficient cells at any time point after IFN priming (Fig. 3e). These experiments suggest that the impact of ISG15 on IFN-α/β-induced signalling may indeed differ between humans and mice.

### Isg15 does not sustain Usp18 levels in mice

**Figure 4:**
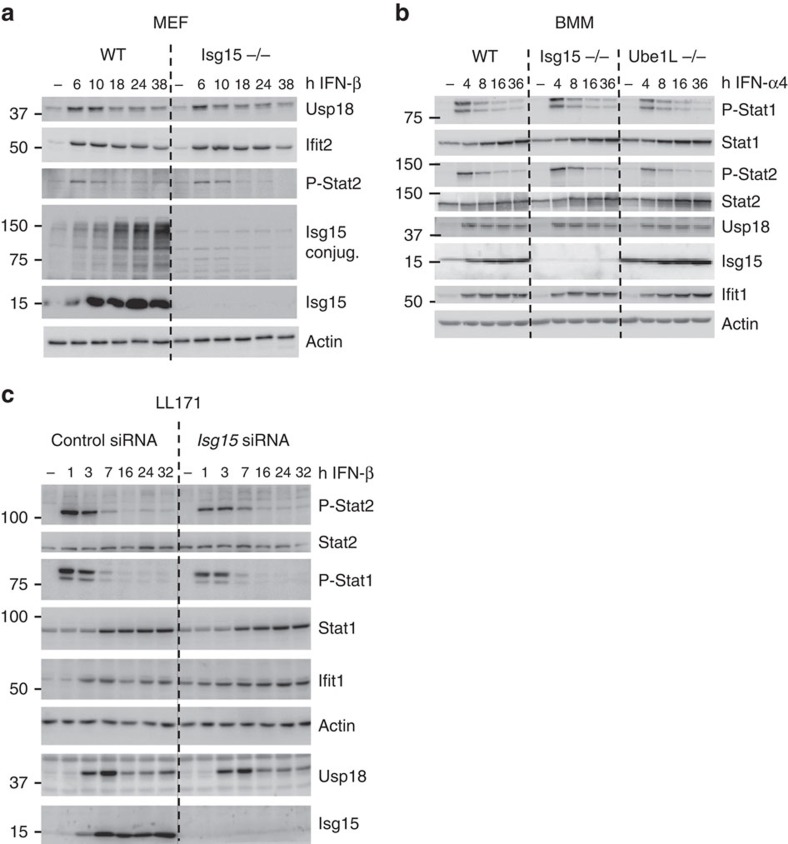
Murine Isg15 does not control Usp18 accumulation and IFN-α/β signalling. (**a**) Primary MEFs from WT and *Isg15*-deficient mice were primed with murine IFN-β (500 pM) for 6–38 h. Cell lysates (30 μg) were analysed by western blotting with the antibodies indicated. (**b**) BMM from WT, *Isg15*-deficient and *Ube1L*-deficient mice were primed with murine IFN-α4 (250 pM) for 4–36 h. Cell lysates (20 μg) were analysed by western blotting with the antibodies indicated. (**c**) Murine LL171 cells were transfected with non-silencing control or ISG15 siRNA. 24 h post-transfection, the cells were primed for 1–32 h with murine IFN-β (10 pM). Cell lysates (30 μg) were analysed by western blotting with the antibodies indicated.

Due to the suboptimal levels of USP18, IFN-α/β signalling persists and ISG transcripts accumulate in *ISG15*-deficient human cells^10^. We, therefore, investigated whether late IFN-α/β signalling and Usp18 levels were also affected in *Isg15*-deficient murine cells. WT and *Isg15*-deficient MEFs were subjected to prolonged stimulation with IFN-β, and the levels of phosphorylated Stat2 and IFN-stimulated proteins were measured by western blotting. In contrast to what was observed for human fibroblasts, WT and *Isg15*-deficient MEFs accumulated similar levels of phosphorylated Stat2, Ifit2 and Usp18 over the time course of the experiment (Fig. 4a). The IFN-α/β response was also studied in primary bone marrow-derived macrophages (BMM) from WT, *Isg15*-deficient or *Ube1L*-deficient (free Isg15 present, but no ISGylation) mice. No major differences were observed between the three strains, indicating that neither free, nor conjugated Isg15 was required to stabilize Usp18 in mice (Fig. 4b). Similar conclusions were drawn from studies of a murine fibroblast cell line (LL171), in which *Isg15* was silenced with siRNA (Fig. 4c). These results provide strong evidence to suggest that, in the mouse system, Isg15 is not involved in the Usp18-mediated negative feedback loop.

### USP18 stabilization by ISG15 is species-specific

**Figure 5 f5:**
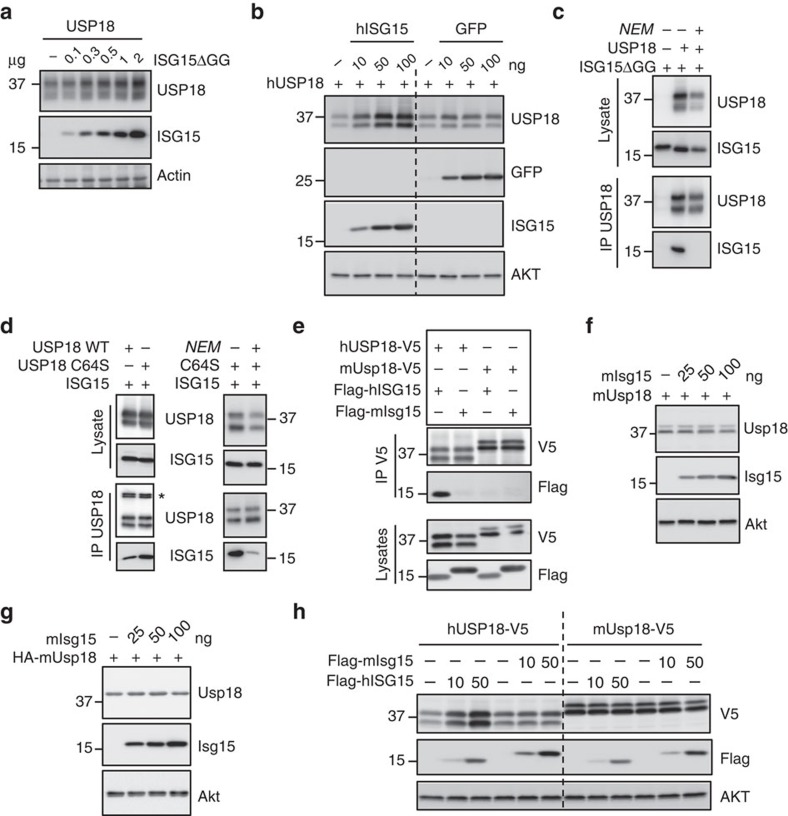
Murine free Isg15 does not interact stably with Usp18. (**a**) HEK293T cells were transfected with a human USP18 expression vector (0.5 μg) alone or with increasing amounts of the human Flag-ISG15ΔGG construct. 48 h post transfection, cell lysates were analysed by western blot with antibodies against USP18 and Flag. (**b**) Cells were transfected with human USP18 (0.5 μg) alone or with increasing amounts of human ISG15 or GFP expression vectors. 48 h later lysates were analysed with the indicated antibodies. (**c**) Cells were transfected with human Flag-ISG15ΔGG (3 μg) alone or with human USP18, as indicated (1.5 μg). NEM (10 mM) was added to the lysis buffer (lane 3). Lysates were subjected to IP with USP18 antibodies. Lysates and co-IP eluates were analysed with antibodies against USP18 and Flag. (**d**) Left panel: cells were transfected with human Flag-ISG15 (1.5 μg), and either human USP18 WT or USP18 C64S (1.5 μg). 48 h later, lysates were subjected to anti-USP18 IP. Lysates and co-IP eluates were analysed with antibodies against USP18 and Flag. Right panel: cells were cotransfected with Flag-ISG15 (1.5 μg) and USP18 C64S (1.5 μg). NEM (10 mM) was added to the lysis buffer (lane 3). Lysates were subjected to anti-USP18 IP. Lysates and co-IP eluates were analysed with antibodies to USP18 and Flag. (**e**) Cells were cotransfected with human or murine USP18-V5 and human or murine Flag-ISG15. 48 h later, lysates were subjected to co-IP with anti-V5 antibodies. Co-IP eluates (top panels) and total lysates (bottom panels) were analysed with the indicated antibodies. (**f**) Cells were transfected with untagged murine Usp18 (0.5 μg) alone or with increasing amounts of murine Flag-Isg15. Lysates were analysed with antibodies to murine Usp18, Flag and Akt. (**g**) As in **f**, except that HA-tagged murine Usp18 was transfected and detected with HA antibodies. (**h**) Cells were transfected with human USP18-V5 or murine Usp18-V5 Usp18 (500 ng) alone or with the indicated amount of human Flag-ISG15 or murine Flag-Isg15. 48 h later, cell lysates were analysed by western blot.

The unique properties of the USP18 or ISG15 orthologues, or of both of them, may underlie the observed species-specific functional differences. ISG15 is weakly conserved across species^34^, whereas the murine and human USP18 proteins are 70.1% identical and 79.7% similar. We confirmed that human ISG15 stabilizes USP18 through ISGylation-independent binding^10^, since the conjugation-deficient ISG15ΔGG mutant was able to stabilize USP18 (Fig. 5a). Furthermore, co-expression of USP18 with an unrelated protein (GFP) did not sustain USP18 levels (Fig. 5b). The ISG15:USP18 complex could be detected by co-immunoprecipitation (co-IP; Fig. 5c, lane 2) and was abolished in the presence of N-ethylmaleimide (NEM), which alkylates cysteine residues (Fig. 5c, lane 3). Yet, the ISG15:USP18 complex retained NEM sensitivity even when we used the USP18-C64S catalytic mutant, suggesting a role for cysteine residues outside of the catalytic core (Fig. 5d).

Next, we tested whether murine Isg15:Usp18 complexes could be detected by co-IP. Weak or completely absent interactions between the two murine proteins was detected in co-IP assays (Fig. 5e). However, USP18 is the cognate deISGylase both in humans and mice. To further examine this interaction we used a micropatterning approach to quantify the stability of the ISG15:USP18 complex in living cells^35^. In this experimental setup, the murine Usp18:Isg15 complex was observed, but it exhibited reduced stability when compared to the human complex (Supplementary Figs 3 and 4, Supplementary Movie 1 and Supplementary Methods). The controls for the micropatterning and fluorescence recovery after photobleaching experiments are outlined in Supplementary Fig. 4. Next, we tested whether transient co-expression of murine Isg15 and Usp18 influenced the levels of murine Usp18. Murine Isg15 failed to sustain the level of murine Usp18 (Fig. 5f–h), in contrast to the phenotype observed with the human orthologues. The inability of overexpressed murine Isg15 to sustain murine Usp18 is consistent with our functional data on the endogenous murine proteins (Fig. 4) and with the comparable downregulation of IFN-α/β responses in WT and *Isg15*-deficient mice *in vivo* (Fig. 3a–d). Altogether, these results indicate that the difference in viral susceptibility between *ISG15*-deficient humans and mice stems from the need for ISG15 to stabilize USP18, and thus downregulate the IFN-α/β response, in humans but not in mice.

## Discussion

IFN-α/β signalling and ISG induction are controlled at multiple levels by negative regulators, such as SOCS (suppressor of cytokine signalling), PIAS (protein inhibitor of activated STAT), protein phosphatases (for example, SHP1) and USP18 (refs 36, 37, 38). However, we have shown that these regulators are not sufficient, and that ISG15 is required for complete downregulation of IFN-α/β signalling (Fig. 1a). Humans lacking ISG15 consistently display high steady-state levels of ISGs in whole blood, and in patient-derived fibroblasts following priming with IFN-α/β (ref. 10). We hypothesized that this dysregulation of IFN-α/β signalling would account for the lack of an overt viral susceptibility in humans, in contrast to what was observed in mice. We show here that, unlike the antiviral activity of Isg15 observed in mice, human ISG15 promotes a proviral state following IFN priming. The persistent ISG expression observed in human *ISG15*-deficient cells imparts a resistance to infection with multiple families of viruses with both RNA and DNA genomes.

These findings contrast from previous reports that have ascribed an antiviral role to ISG15 in human cells. Shi *et al*.^29^ reported that, following silencing of ISG15 for 48 h in HEK-293 cells, SeV titres increased by ∼0.5 and ∼1.0 log at 6 h and 12 h post infection, respectively. Tang *et al*.^23^ found that following ISG15 silencing, A549 cells became ∼fivefold more susceptible to infection by IAV (A/PR/8/1934). While these studies point to an antiviral role of ISG15 in human cells, our experiments with SeV and IAV do not capture the small observed difference in viral growth when comparing WT and *ISG15*-deficient hTert-immortalized fibroblasts without IFN priming (Fig. 1). These studies focused on early timepoints of the acute IFN response induced by the virus, and did not capture the more powerful role of ISG15 as a negative regulator of IFN signalling. Even if there may be a small antiviral role for ISG15 during the acute IFN response, it is clinically irrelevant *in vivo* and not readily captured *in vitro.*

Interestingly, we observed that the conjugation-deficient ISG15ΔGG was as efficient as WT ISG15 in rescuing viral replication in *ISG15*-deficient patient cells. One could have predicted an even better rescue of both the ISG expression and infection phenotypes by ISG15ΔGG, since the latter should be more readily accessible to mediate USP18 stability than the conjugation-competent form of ISG15. However, this was not the case, suggesting the possibility that ISG15ΔGG may have lower USP18-binding affinity or that only a small amount of ISG15 is required to stabilize USP18, and the increased availability of ISG15ΔGG has no further effect. An additional possibility, albeit less likely given the depth of work demonstrating the antiviral role of ISGylation, is that ISG15 in the conjugated form may also exhibit some proviral properties.

We have shown that free ISG15 associates with higher affinity to USP18 in humans as compared with mice, although the species-specific structural determinants involved in complex formation have yet to be identified. Another species-restricted complex involving free ISG15 has been described. The NS1 protein of influenza B virus binds non-covalently to human and non-human primate ISG15, but not to murine Isg15 (refs 39, 40, 41). Mutational and structural studies of the NS1:ISG15 complex have shown that the molecular determinant of the binding specificity (humans versus mice) lies in the small hinge region of ISG15 (refs 42, 43). Further work is required to identify the domain(s) responsible for the human ISG15:USP18 interactions that are required to enhance stability and properly regulate the IFN responses.

Unlike humans, mice can downregulate IFN signalling regardless of ISG15 competency and it is currently unknown whether murine Usp18 is stabilized by another protein, IFN-stimulated or otherwise. Individuals lacking ISG15 exhibit elevated ISG expression, which may confer an enhanced protection against viral infection, but do not exhibit any of the side-effects associated with IFN treatment^10^. As such, the USP18:ISG15 interaction is a potentially attractive target for small molecule-based short-term treatments to boost endogenous IFN activity.

Our findings raise interesting questions about the evolutionary role of ISG15. Given the detrimental effects that inflammation may cause in species with a longer lifespan, gain-of-function binding mutations in ISG15 and/or USP18 may reflect an evolutionary necessity in humans, and likely other mammals, to more stringent tuning of the IFN system. On the other hand, viruses have developed immune evasion strategies in parallel. Indeed, many human viral pathogens were shown to oppose ISGylation (influenza B virus NS1^39^ and vaccinia virus E3L^16^,^44^ proteins) or induce de-ISGylation (numerous viral L proteins containing ovarian tumour^45^ or papain-like protease^46^). Much of the work characterizing these viral proteins has focused on the inhibition/removal of conjugated ISG15 from viral and host targets. However, in light of our data demonstrating that the primary role of human ISG15 is to stabilize USP18 and downregulate the IFN response, it may be that inhibition of ISGylation and removal of conjugated ISG15 is a novel mode of IFN antagonism. By enhancing the pool of free ISG15, these viral proteins may enhance USP18 levels, dampen IFN signalling and promote viral escape.

Based on current knowledge, murine Isg15 acts in an antiviral manner (largely *via* ISGylation), while murine Usp18 functions in a proviral manner (IFN negative regulation and de-ISGylation). By contrast, human ISG15 and USP18 both appear to function in an anti-inflammatory manner. Consequently, this makes them proviral, although the ISGylation of cellular or viral proteins may play a non-dominant antiviral role. However, if both ISGylation and de-ISGylation are proviral (through provision of the free ISG15 required for USP18 stabilization), this notion becomes somewhat paradoxical. In humans then, this process may serve as a mechanism for the temporal regulation of IFN-α/β-induced signalling. We suggest that one of the roles of ISGylation at early stages of viral infection in humans is to sequester free ISG15, thereby allowing IFN signalling to occur. At later stages, USP18-mediated de-ISGylation releases free ISG15, which in turn stabilizes USP18 and tunes down IFN-α/β signalling and inflammation.

## Methods

### Cells

Icahn School of Medicine IRB board has approved the use of human subject cell lines. Dermal fibroblasts from *ISG15*-deficient patients (*n*=3) and WT controls (*n*=5) were immortalized by stable transduction with hTert^10^. Patient (*n*=3) and control (*n*=2) cells were stably complemented with ISG15, ISG15ΔGG or luciferase by lentiviral transduction^10^. Transduced fibroblasts were sorted on the basis of RFP expression (BD FACS Aria II). The stable expression of the transduced constructs was periodically checked by western blotting. Murine embryonic fibroblasts from WT C57BL6/J and *Isg15*-deficient mice were derived by disaggregation of day 13.5 embryos from timed matings by trituration of the embryos at 37 °C in trypsin^19^. Mouse bone marrow macrophages (BMM) were generated by isolating bone marrow cells capable of adhering to non-tissue-culture treated plates for 7 days in BMM media (Dulbeco's Modified Eagle Medium, 10% fetal calf serum, 5% horse serum, 2% supernatant derived from CMG14-12 cells)^47^. LL171 cells (clonal isolate of murine L929 fibroblasts; from G. Uzé, CNRS, Montpellier, France), HEK293T (CRL-11268; ATCC), Vero-E6 (CRL-1586; ATCC), Vero (CCL81; ATCC), HeLa (CCL-2; ATCC), A549 (CCL-185; ATCC), murine embryonic fibroblasts and human fibroblasts were cultured in normal growth medium consisting of DMEM supplemented with 10% fetal calf serum. All cells were cultured at 37 °C and 10% CO_2_. All cells were tested for mycoplasma contamination using the PlasmoTest kit according to the manufacturer instructions (Invivogen).

### Priming

Untransduced, luciferase-, ISG15-, and ISG15ΔGG-transduced fibroblasts (Figs 1, 2, 3) were primed by incubation with 0, 10, 100, or 1,000 IU ml^−1^ IFN-α2b (Merck IntronA) in normal growth medium for 12 h. The IFN-α2b was then eliminated by thorough washing with PBS and the cells were allowed to rest for 36 h in normal medium before infection. Murine embryonic fibroblasts were primed with 1,000 U universal type-I IFN (Fig. 3; PBL IFN) or 500 pM murine IFN-β (Fig. 4; PBL IFN). Mouse bone marrow macrophages were primed with 250 pM murine IFN-α4 (Fig. 4; Calbiochem). LL171 cells were primed with 10 pM murine IFN-β (Fig. 4; PBL IFN).

### ISG Expression analysis

RNA was extracted from fibroblasts (Qiagen RNeasy) or whole blood (PAXgene Blood RNA Kit) and reverse-transcribed (ABI High Capacity Reverse Transcriptase) according to the instructions provided by the manufacturer. Levels of ISG expression (*IFIT1, MX1*), relative to the *18S* rRNA housekeeper gene, were analysed by Taqman quantitative real-time PCR (TaqMan Universal Master Mix II w/ UNG, Roche LightCycler 480 II). The relative expression levels of the ISGs were calculated by the ΔΔCT method, with comparison with the mean value for mock-treated controls.

### Viral infections

All infections were performed 48 h after IFN treatment (12 h priming, 36 h rest) in normal growth medium. The viruses and multiplicities of infection used were as follows: VSV Indiana strain at an MOI of 1.0, rVSV–GFP Indiana strain^48^ at an MOI of 10.0, rHSV-1 US11-GFP Patton strain^49^ at an MOI of 1.0 (kindly provided by the laboratory of Ian Mohr), rHCMV IE2-YFP strain AD169 (ref. 50) at an MOI of 2.0, rIAV A/PR/8/34 gLuc virus^51^ modified by the introduction of a PTV1 2A cleavage site (PR8-gLuc-PTV1; kindly provided by Nick Heaton) at an MOI of 10.0, rSeV-eGFP Fushimi strain^52^ modified by the introduction of a gLuc-P2A cleavage site (rSeV-gLuc-P2A-eGFP; same method as for NiV below) at an MOI of 0.1, RVFV strain ZH501 at an MOI of 0.01, and the rNiV-gLuc-P2A-eGFP Malaysian strain^52^ at an MOI of 0.01. All work with infectious RVFV and rNiV was carried out under biosafety level 4 (BSL-4) conditions at the Robert E. Shope BSL-4 laboratory at the University of Texas Medical Branch.

### Analysis of viral replication

VSV-infected supernatants were removed 24 h.p.i. and titered by determining the 50% tissue culture infectious dose on Vero-E6 cells. RVFV and rNiV-gLuc-P2A-eGFP-infected supernatants were collected at 48 and 24 h.p.i., respectively, and titered by plaque assays on Vero-CCL81 cells. Fibroblasts infected with rVSV–GFP or rHSV-1-US11-GFP were fixed in 4% PFA 24 h.p.i. and stained with 5 μg ml^−1^ Hoechst 33342 in PBS. Cells were imaged and the results were analysed (BioTek Cytation 3; Gen5 software). HCMV-IE2-YFP-infected cells were fixed with 4% PFA 24 h.p.i., and imaging analysis was performed on an Acumen Explorer plate cytometer. Fibroblasts infected with IAV PR8-gLuc-PTV1 were collected and washed with PBS at 24 h.p.i. The cells were lysed and assayed for luciferase activity, according to the manufacturer's instructions (Promega Renilla Luciferase Assay System; BioTek Cytation 3). We collected rSeV-gLuc-P2A-eGFP-infected supernatants were collected at 24 h.p.i. Supernatant samples were inactivated with lysis buffer, and assayed for luciferase activity according to the manufacturer's instructions (Promega Renilla Luciferase Assay System; BioTek Cytation 3).

### Mouse studies

Co-housed 10-week-old female C57BL6/J (Jackson) and *Isg15*-deficient^19^ mice received intraperitoneal injections of PBS or 10,000 IU universal type-I IFN (PBL Interferon Source). At the post-treatment times indicated, the mice were sacrificed and exsanguinated for the analysis of ISG levels in whole blood. All procedures were carried out in accordance with National Institutes of Health and Icahn School of Medicine at Mount SinaiInstitutional Animal Care and Use Committee guidelines.

### Plasmids and transfection

HEK-293 T cells were transiently transfected with Lipofectamine-2000 (Invitrogen), complexed with the following constructs according to the instructions provided by the manufacturer. Pseudotyped lentiviral particles were produced by transfection of pCAGGS-VSV-G, pCMV-Gag/Pol and pTRIP-hISG15ΔGG-IRES-RFP. The following constructs were used for expression and co-IP assays: pcDNA4b-huUSP18 (ref. 53), pcDNA3-3xFlag-huISG15 (from J. Huibregtse, University of Texas at Austin, TX, USA), pBabe-3xFlag-His6-huISG15ΔGG was derived from the construct above; pBK-CMV-mUsp18, pcDNA3-HA-mUsp18 and pCAGGS-Flag-mIsg15 (Addgene), pEGFP-N1 (Clontech), pcDNA4B-hUSP18-V5, and pcDNA4B-mUsp18-V5.

### siRNA-mediated silencing

Murine LL171 cells were silenced by incubation for 24 h with 25 nM Isg15 siRNA (5′-CACAGUGAUGCUAGUGGUACA-3′; Sigma) followed by priming with IFN. HeLa and A549 cells were silenced by incubation for 24 h with 25 nM ISG15 siRNA followed by priming with IFN. siRNA transfections were performed with Lipofectamine RNAi max reagent (Invitrogen) according to the manufacturer's instructions.

### Protein analysis

Cells were lysed in radioimmunoprecipitation assay buffer (RIPA buffer; 10 mM Tris-Cl, 1 mM EDTA, 0.5 mM EGTA, 1% Triton X-100, 0.1% sodium deoxycholate, 0.1% SDS, 140 mM NaCl, 150 mM PMSF, protease inhibitor cocktail (Sigma-Aldrich), 1 μg ml^−1^ okadaic acid (Merck), and Halt phosphatase inhibitor (Thermo Scientific)) and analysed by western blot. For co-IP assays, transfected cells were lysed in 50 mM Tris pH 6.8, 0.5% Nonidet P40, 200 mM NaCl, 10% glycerol, 1 mM EDTA and a protease inhibitor cocktail. Sensitivity to NEM (Sigma-Aldrich) was assessed by adding this compound to the lysis buffer at a concentration of 10 mM. USP18 was immunoprecipitated with anti-human USP18 (Cell Signaling Technology D4E7, 1:250) or anti-V5 (Invitrogen R960-25, 1:200) monoclonal antibodies. The following antibodies were used for western blot analysis: human ISG15 (Santa Cruz 3E5, 1:1,000; ABGENT AP1150a, 1:1,000; mAb cl. 2.1, 1:1,000 (a gift from E.C. Borden, Cleveland Clinic, Cleveland OH^54^)); human USP18 (Cell Signaling Technology D4E7, 1:5,000); murine Isg15 (rabbit mAb 1551, 1:3,000; or rabbit pAbs, 1:5,000 (a gift from A.L. Haas, LSU Health Sciences Center School of Medicine, New Orleans, LA^55^); murine Usp18 (1:3,000 (a gift from K.P. Knobeloch, University of Freiburg, Freiburg, Germany^56^); V20 (Santa Cruz Biotechnology sc-50019, 1:200); murine Ifit1 (1:5,000) and Ifit2 (1:10,000; gifts from G. Sen (Cleveland Clinic, Cleveland, OH^57^); Stat1 (Millipore 06-501, 1:2,000); phospho-Stat1 (Cell Signaling Technology 9171 L, 1:1,000); murine Stat2 (1:5,000) (a gift from C. Schindler, Columbia University, NY, NY^58^); phospho-Stat2 (Millipore 07224, 1:1000); AKT (Cell Signaling Technology 40D4, 1:1000); actin (Sigma-Aldrich cl. AC-40, 1:10,000); HA (Sigma-Aldrich H9658, 1:2,000); Flag (Sigma-Aldrich F3165, 1:5,000); GFP (Rockland Immunochemicals 600-101-215, 1:1,000); V5 (Sigma-Aldrich V8137, 1:5,000). Antibody binding was detected by enhanced chemiluminescence (Western Lightning, Perkin Elmer). Relative band intensities were determined with a Fuji ImageQuant LAS-4000. Uncropped images for Fig. 4a and b can be found in Supplementary Fig. 5a–b. Uncropped images for Fig. 5a,e,f,h, [Fig f5], [Fig f5], [Fig f5] can be found in Supplementary Fig. 6a–d (ref. 59).

## Additional information

**How to cite this article:** Speer, S. D. *et al*. ISG15 deficiency and increased viral resistance in humans but not mice. *Nat. Commun.* 7:11496 doi: 10.1038/ncomms11496 (2016).

## Supplementary Material

Supplementary Information Supplementary Figures 1-6, Supplementary Methods and Supplementary References

Supplementary Movie 1 FRAP of SiRSNAPf-mISG15 bound to mEGFP-mUSP18 Scale bar: 10 μm. Dynamic video demonstrating USP18 and ISG15 interactions in the micro patterning system as described for Supplementary Figure 3 and 4.
