# Supplementary material for: ISG15 deficiency and increased viral resistance in humans but not mice
Source: Nat Commun. 2016 May 19;7:11496. doi: 10.1038/ncomms11496 (PMC4873964; doi:10.1038/ncomms11496)
Supplement: Supplementary Information — Supplementary Figures 1-6, Supplementary Methods and Supplementary References [file ncomms11496-s1.pdf]

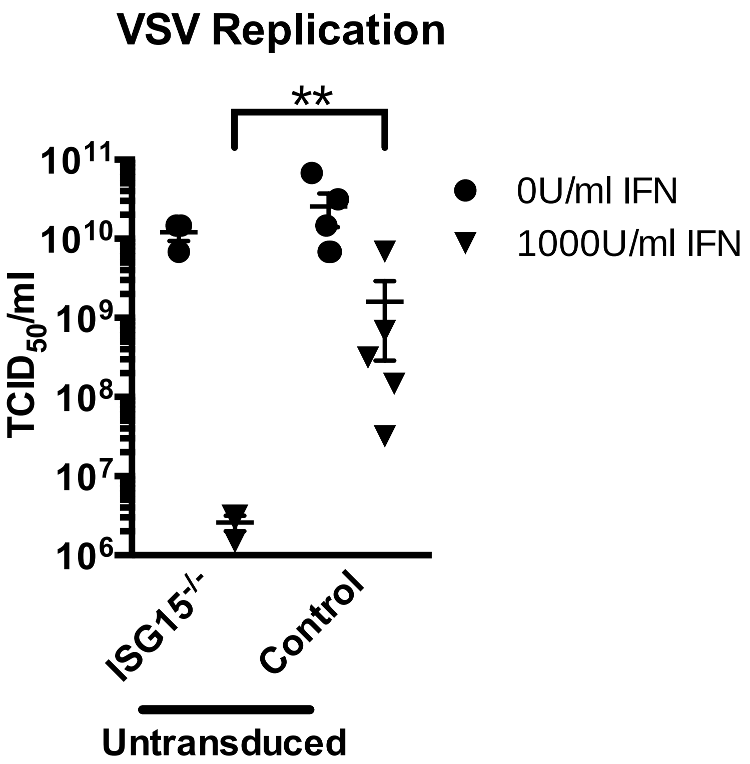

Supplementary Figure 1 - *ISG15*-deficient cells exhibit resistance to infection with VSV

hTert-immortalized fibroblasts from *ISG15*-deficient patients (n=3) or controls (n=5) were treated with the indicated concentration of IFN-α2b for 12 h, washed, and allowed to rest for 36 h before infection. Cells were infected with VSV at an MOI of 1.0 for 24 h. Supernatants were titered by TCID50. Shown is a single experiment of three performed. Error bars = SD. Comparisons made with unpaired t-test. **p<0.01.

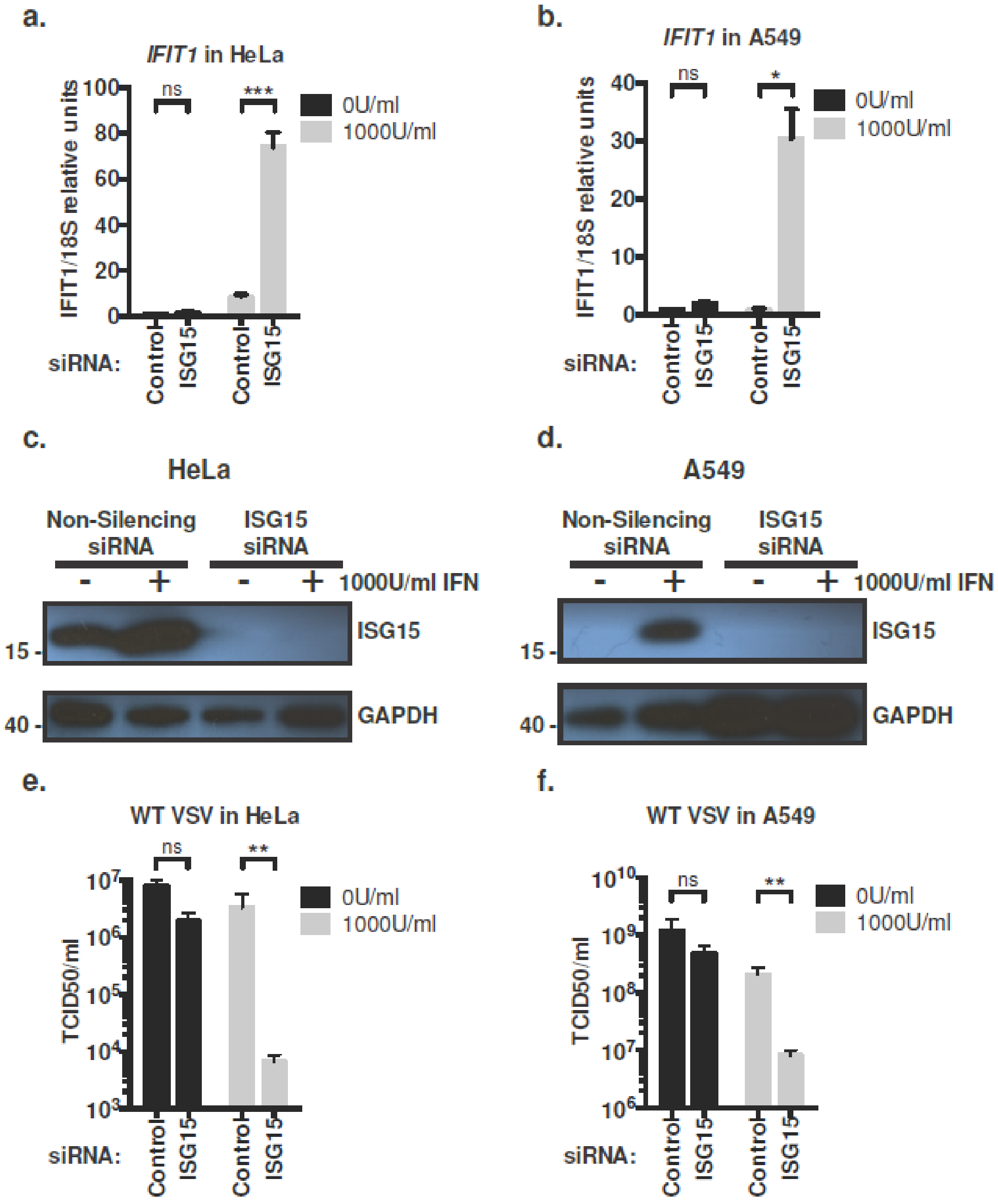

Supplementary Figure 2 - siRNA knockdown of ISG15 in HeLa and A549 results in enhanced ISG expression and resistance to VSV infection

(**a**)**,** (**c**) and (**e**) HeLa cells and (**b**)**,** (**d**) and (**f**) A549 cells were transfected with siRNA targeting ISG15 or a non-silencing control and incubated for 24 h. The cells were then treated with 0 or 1000 IU ml^{-1} IFN-α2b for 12 h, washed, and allowed to rest for 36 h before infection. Cell lysates were collected 48 h post-priming. Relative *IFIT1* mRNA expression levels were quantified by qPCR for (**a**) HeLa and (**b**) A549 cells. siRNA knockdown efficiency in (**c**) HeLa and (**d**) A549 cells was analyzed by western blot using antibodies against ISG15 and GAPDH. (**e**) HeLa and (**f**) A549 cells were infected with VSV at an MOI of 1.0 for 24 h. Supernatants were titered by TCID50. Panels (**a**), (**b**), (**e**), and (**f**) show the combined results of two experiments. Panels (**c**) and (**d**) show a single representative blot of two performed. Error bars = SD. Comparisons made with unpaired t-test. ns=not significant, *$p<0.05$, **$p<0.01$, ***$p<0.001$.

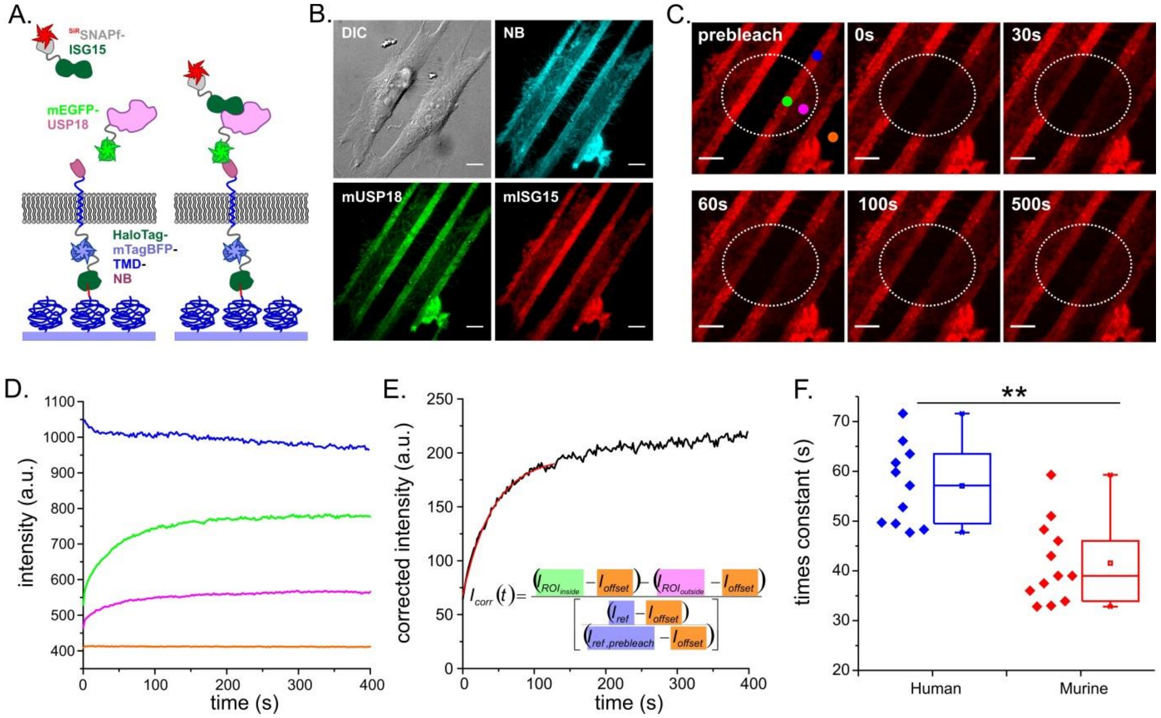

Supplementary Figure 3 - Quantifying the stability of the ISG15:USP18 complex in living cells

(**a**) Cartoon depicting micropatterning of the USP18:ISG15 complex. Hela cells expressing a transmembrane domain (TMD) fused to the HaloTag and mTagBFP at the extracellular N-terminus and an anti-GFP nanobody at the intracellular C-terminus (HaloTag-mTagBFP-TMD-NB) were cultured on cover slides presenting micropatterned HaloTag ligand (HTL, red). Upon coexpression of USP18 fused to mEGFP (mEGFP-USP18) and ISG15 fused to SNAPf (SNAPf-ISG15), capturing of USP18-ISG15 complexes into micropatterns is observed. (**b**) Typical experiment for the mUSP18-mISG15 complex showing two cells co-expressing HaloTag-mTagBFP-TMD-NB (cyan), mEGFP-mUSP18 (green) and SNAPf-mISG15 stained with BG-SiR (red). Scale bar: 10 µm. (**c**) Fluorescence recovery after photobleaching (FRAP) experiment monitoring the exchange of bleached SiRSNAPf-mISG15 in micropatterns by unbleached SiRSNAPf-

mISG15 from the cytosol (cf. movie S1). Scale bar: 10 µm. (**d**) Typical FRAP curves obtained in different regions, color-coded as depicted in the first image shown in (c). (**e**) FRAP curve corrected for background and photobleaching[1] according to the equation shown in the inset (same color coding as in c and d) and exponential fit (red line). (**f**) Box plots of complex lifetimes obtained for the human and the murine USP18/ISG15 complexes with each dot corresponding to an independent FRAP experiment. Mean complex lifetimes of (41 ± 8) s and (57 ± 8) s were obtained for the murine and the human complex, respectively ($p=0.0012$).

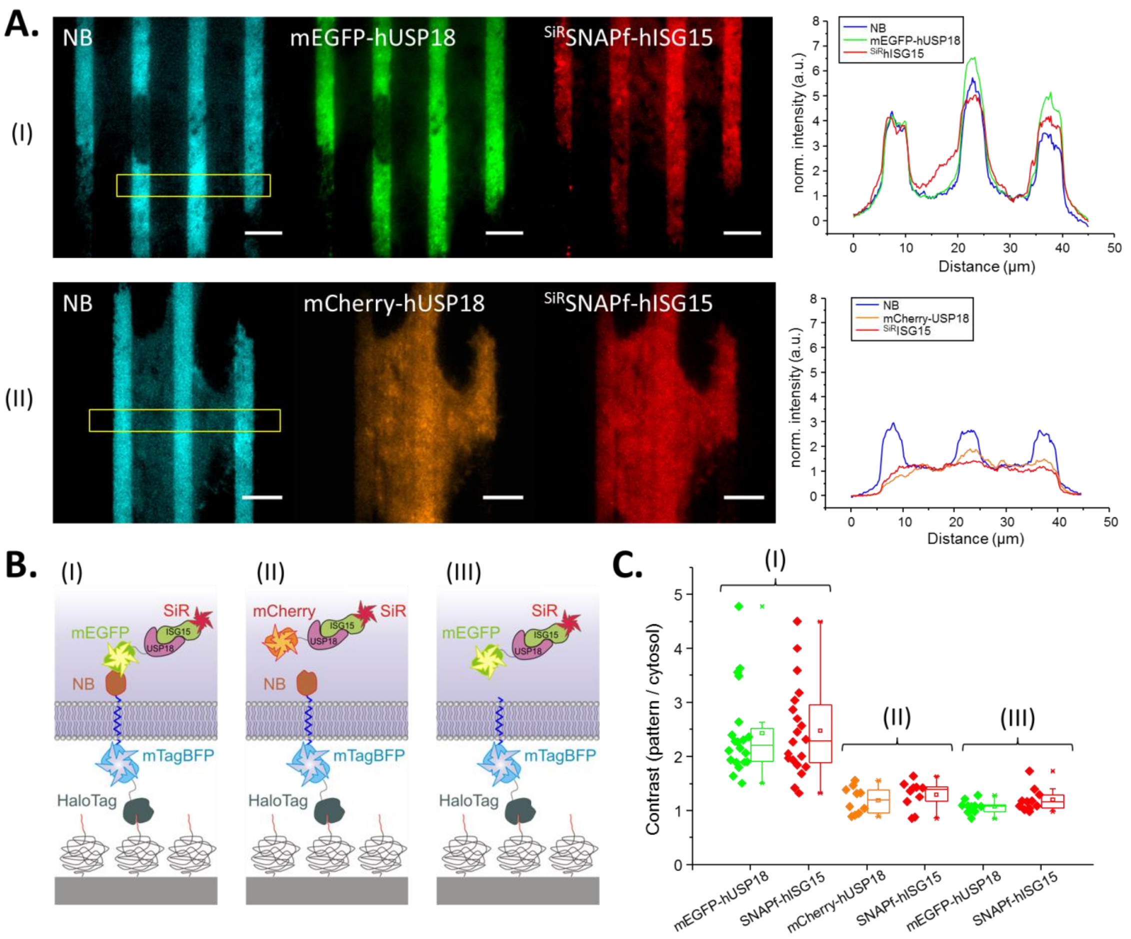

Supplementary Figure 4 - Controls confirming specificity of micropatterning assays

(**a**) HeLa cells expressing anti-GFP NB, mEGFP-hUSP18 and SNAPf-hISG15 (**I**) compared to HeLa cells expressing anti-GFP NB, mCherry-hUSP18 and SNAPf-hISG15 (**II**). Intensity profiles across the micropatterned region highlighted by the yellow boxes are overlayed in the diagrams for all three channels of each experiment. (**b**) Cartoon depicting the binding experiment (**I**) and negative controls (**II**, **III**). (**c**) Box plot showing the contrast of binding experiment (**I**) and negative controls (**II**, **III**) according to the cartoons shown in B. Each dot corresponds to a single cell. A contrast of 1 (indicated by the dotted line) corresponds to the same intensity inside and outside the functionalized region.

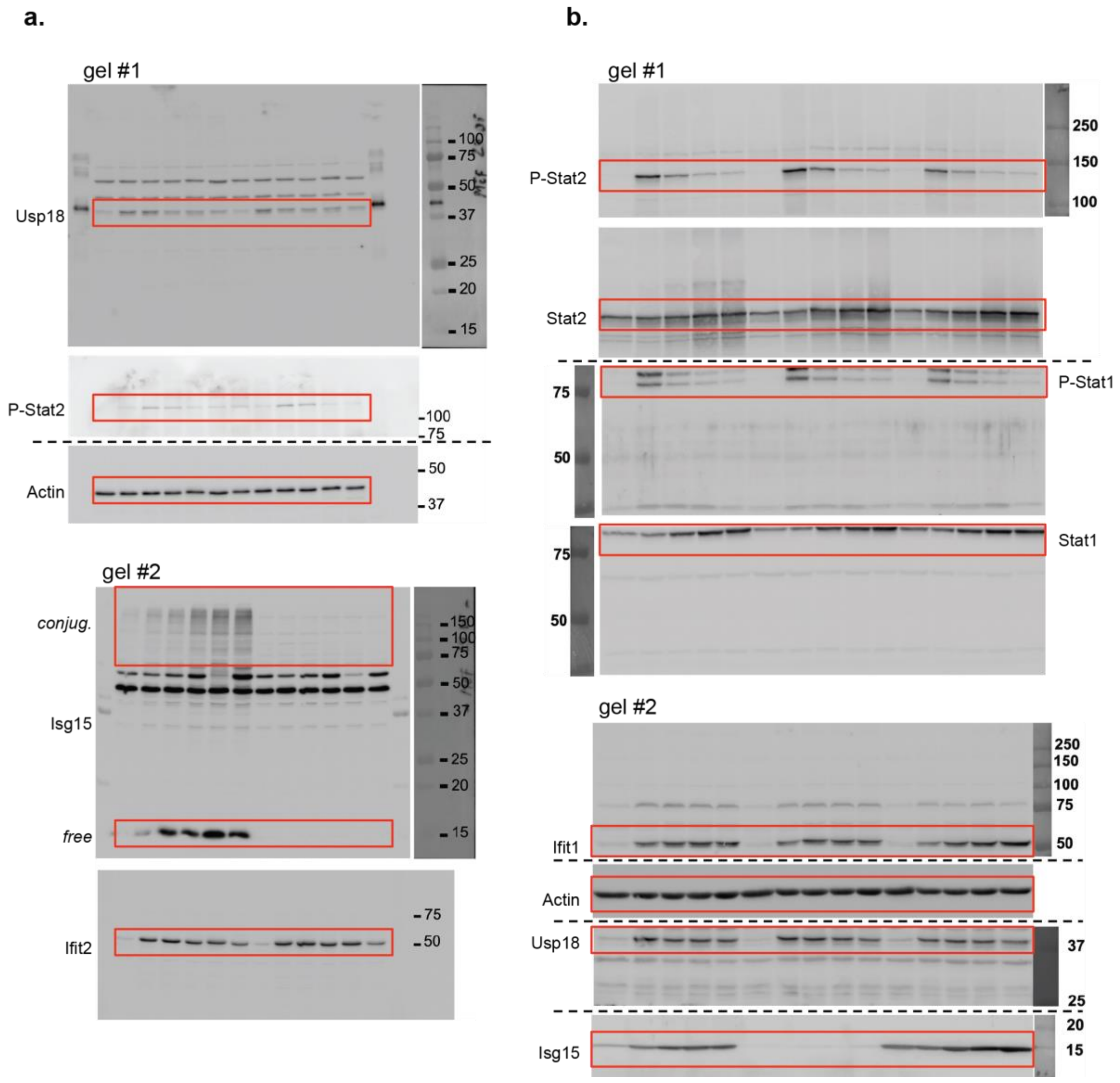

Supplementary Figure 5 - Murine Isg15 does not control Usp18 accumulation and IFN-α/β signaling

Uncropped blots from Fig. 4. (**a**) Primary MEFs from WT and *Isg15*-deficient mice were primed with murine IFN-β (500 pM) for 6-38 h. Cell lysates (30 µg) were analyzed by western blotting with the antibodies indicated. (**b**) BMM from WT, *Isg15*-deficient and *Ube1L*-deficient mice were primed with murine IFN-α4 (250 pM) for 4-36 h. Cell lysates (20 µg) were analyzed by western blotting with the antibodies indicated.

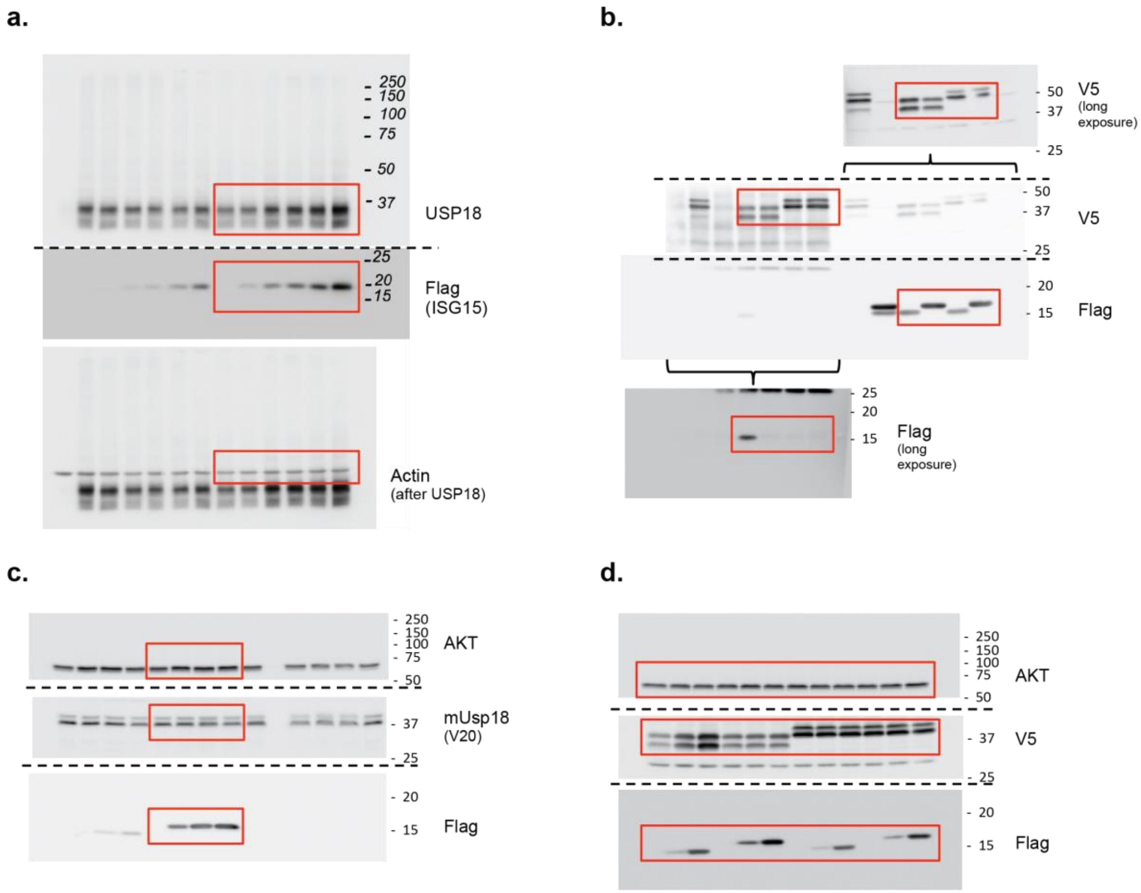

Supplementary Figure 6 - Murine free Isg15 does not interact stably with Usp18

Uncropped blots from Fig. 5. (**a**) HEK293T cells were transfected with a human USP18 expression vector (0.5 µg) alone or with increasing amounts of the human Flag-ISG15ΔGG construct. 48 h post-transfection, cell lysates were analyzed by western blot with antibodies against USP18 and Flag. (**b**) Cells were cotransfected with human or murine USP18-V5 and human or murine Flag-ISG15. 48 h later, lysates were subjected to co-IP with anti-V5 antibodies. Co-IP eluates (top panels) and total lysates (bottom panels) were analyzed with the indicated antibodies. (**c**) Cells were transfected with untagged murine Usp18 (0.5 µg) alone or with increasing amounts of murine Flag-Isg15. Lysates were analyzed with antibodies to murine Usp18, Flag and Akt. (**d**) Cells were transfected with human USP18-V5 or murine Usp18-V5 Usp18 (500 ng) alone or with

the indicated amount of human Flag-ISG15 or murine Flag-Isg15. 48 h later, cell lysates were analyzed by western blot.

Supplementary Methods

Live cell micropatterning - For micropatterning HaloTag fusion proteins in the plasma membrane of live cells, glass coverslips were micropatterned functionalized with HTL by microcontact printing of poly-L-lysine-graft-poly (ethylene glycol) (PLL-PEG) functionalized with HaloTag ligand and backfilling with PLL-PEG functionalized with an RGD peptide[2]. For capturing cytosolic protein complexes into HTL micropatterns, the HaloTag fused to mTagBFP, a transmembrane domain (TMD) and an anti-GFP Nanobody (HaloTag-mTagBFP-TMD-NB) were cloned into the pDisplay vector (Invitrogen). As bait proteins, human USP18 and murine USP18, respectively were fused to an N-terminal mEGFP (mEGFP-mUSP18 and mEGFP-hUSP18, respectively) using the pSEMS-26m vector backbone. Human ISG15 and murine Isg15, respectively, were fused to an N-terminal SNAPf (SNAPf-ISG15) and cloned into the pSEMS-26m vector by cassettes cloning as described before. HeLa cells were transiently co-transfected with the three plasmids and cultivated on micropatterned support[1]. Prior to imaging, HeLa cells were labeled with 100 nM SNAP-Cell TMR-STAR or 100 nM BG-SIR for 30 minutes. Fluorescence imaging of transiently transfected HeLa cells was carried out with an inverted TIRF microscope (Olympus Cell^TIRF Xcellence) equipped with lasers for excitation at 405 nm (mTagBFP), 488 nm (mEGFP), 561 nm (TMR) and 640 nm (SiR). A 60× objective with an NA of 1.45 (U Apochromat N 60×/1.45 NA; Olympus) was applied for TIR excitation. Imaging and FRAP experiments were carried out at room temperature (25 °C) with cells kept under MEM medium[1]. For determining complex lifetimes, FRAP curves were fitted by a monoexponential function[1].

Supplementary References

1. Lochte, S., Waichman, S., Beutel, O., You, C. & Piehler, J. Live cell micropatterning reveals the dynamics of signaling complexes at the plasma membrane. *J Cell Biol* **207**, 407-18 (2014).
2. Wedeking, T. *et al.* Spatiotemporally Controlled Reorganization of Signaling Complexes in the Plasma Membrane of Living Cells. *Small* (2015).
